# Maternal perinatal depressive symptoms and oppositional-defiant disorder in children and adolescents

**DOI:** 10.1192/j.eurpsy.2021.592

**Published:** 2021-08-13

**Authors:** B. Dachew, J. Scott, G. Ayano, R. Alati

**Affiliations:** 1 School Of Public Health, Curtin University, Perth, Australia; 2 Berghofer Medical Research Institute, QIMR, Brisbane, Australia

**Keywords:** Perinatal depresssion, oppositional-defiant disorder, ALSPAC

## Abstract

**Introduction:**

There is evidence that maternal perinatal depression is associated with adverse neurodevelopmental and mental health outcomes in children. No study has yet examined the association between maternal depressive symptoms during pregnancy and the postpartum period and the risk of oppositional-defiant disorder (ODD) in children and adolescents.

**Objectives:**

This study aimed to investigate whether there is an association between perinatal depressive symptoms and the risk of ODD in offspring from age 7 to 15 years.

**Methods:**

We used data from the Avon Longitudinal Study of Parents and Children (ALSPAC), a population-based prospective birth cohort study in the UK. Offspring ODD at the age of 7, 10, 13 and 15 years were assessed by using parental reports the Development and Well-Being Assessment (DAWBA). We applied Generalized Estimating Equation (GEE) modelling to examine associations across the four time points.

**Results:**

Maternal postnatal depressive symptoms were associated with more a two-fold increased risk of ODD overall. Third trimester depressive symptoms (measured at 32 weeks of gestation) increased risk of ODD by 72%. Offspring of mothers who had depressive symptoms both during pregnancy and in the first year of postpartum period have a four-fold increased risk of ODD over time (_adjusted_ OR = 3.59 (1.98-6.52).

**Conclusions:**

Offspring of mothers with perinatal depressive symptoms are at an increased risk of developing behavioural disorders.

